# Role of Central Inflammatory and Oxidative Pathways in the Morphine Exacerbation of Cardiovascular Effects of Sepsis in Rats

**DOI:** 10.3390/ph18060882

**Published:** 2025-06-12

**Authors:** Mohamed Abdelnaby, Marwa Y. Sallam, Mai M. Helmy, Hanan M. El-Gowelli, Mahmoud M. El-Mas

**Affiliations:** 1 Department of Pharmacology and Toxicology, Faculty of Pharmacy, Alexandria University, Alexandria 21511, Egypt; abdelnaby9412@gmail.com (M.A.); marwa.salamm@alexu.edu.eg (M.Y.S.); mai.helmy@alexu.edu.eg (M.M.H.); dr_hanan_el_gowali@hotmail.com (H.M.E.-G.); 2 Department of Pharmacology and Toxicology, Faculty of Medicine, Kuwait University, Jabriya 46300, Kuwait

**Keywords:** sepsis, morphine, cardiovascular, autonomic function, inflammation, oxidative stress

## Abstract

**Background/Objectives:** Sepsis remains one of the most serious and possibly fatal complications encountered in intensive care units. Considering the frequent use of narcotic analgesics in this setting, we investigated whether the cardiovascular and peripheral and central inflammatory features of sepsis could be modified by morphine. **Methods:** Rats were instrumented with femoral and intracisternal (i.c.) indwelling catheters and sepsis was induced by cecal ligation and puncture (CLP). **Results:** The i.v. administration of morphine (3 and 10 mg/kg) significantly and dose-dependently aggravated septic manifestations of hypotension and impaired cardiac autonomic activity, as reflected by the reductions in indices of heart rate variability (HRV). Cardiac contractility (dP/dtmax) was also reduced by morphine in septic rats. The morphine effects were mostly eliminated following (i) blockade of μ-opioid receptors by i.v. naloxone and (ii) inhibition of central PI3K, MAPK-ERK, MAPK-JNK, NADPH oxidase (NADPHox), or Rho-kinase (ROCK) by i.c. wortmannin, PD98059, SP600125, diphenyleneiodonium, and fasudil, respectively. Further, these pharmacologic interventions significantly reduced the heightened protein expression of toll-like receptor 4 (TLR4) and monocyte chemoattractant protein-1 (MCP1) in brainstem rostral ventrolateral medullary (RVLM), but not cardiac, tissues of CLP/morphine-treated rats. **Conclusions:** Morphine worsens cardiovascular and autonomic disturbances caused by sepsis through a mechanism mediated via μ-opioid receptors and upregulated central inflammatory, chemotactic, and oxidative signals. Clinical studies are warranted to re-affirm the adverse cardiovascular interaction between opioids and the septic challenge.

## 1. Introduction

Sepsis is a potentially fatal hyperinflammatory illness that follows severe and uncontrolled infection [1]. It is one of the most frequent complications faced in intensive care units (ICUs) [2] and a common cause of death in hospitalized patients [2]. The hallmark characteristics of sepsis are hypotension, impaired HRV and cardiac autonomic activity, end organ failure, encephalopathy and death [3,4,5]. Patho-physiologically, sepsis initiates an immune response and release of proinflammatory cytokines, such as tumor necrosis factor-alpha (TNF-α) and interleukins. This initial response is followed by the production of secondary inflammatory effectors, such as arachidonic acid metabolites, nitric oxide and monocyte chemoattractant protein 1 (MCP-1) [6,7]. All these mediators play a key role in the hypotension and cardiac autonomic dysfunction seen during sepsis [8,9]. Moreover, toll like receptor-4 (TLR4) and its signaling pathway is essential to the activation of the septic proinflammatory response [10]. The peripherally released proinflammatory signals are then actively transported to the brain through the blood–brain barrier or afferent nerves modulation [11,12,13]. The same set of proinflammatory cytokines is then expressed by the innate immune brain cells and contributes to the systemic damaging effect of sepsis [11].

Opioid analgesics are widely and routinely used in ICUs for severe pain control [14]. Reported clinical and experimental studies mostly suggest a detrimental effect for opioids on septic manifestations. The use of opioids in septic patients has been shown to prolong hospitalization and increase mortality [15,16]. Experimentally, tramadol, but not fentanyl, accelerates the course and severity of sepsis-induced mortality in CLP rats [17]. Morphine increases mortality rates in a murine model of polymicrobial sepsis via enhancing the gut translocation of gram-positive bacteria [18]. Moreover, morphine suppresses endotoxin tolerance and accelerates the progression of sepsis and septic shock in mice [19].

The interaction of opioids with cardiovascular sequels of sepsis has been reported in few studies. For example, patients with terminal sepsis exhibit greater falls in blood pressure when treated with opioids [20]. Moreover, fentanyl or tramadol abolishes the hypotensive response to sepsis in CLP rodents [17]. In the current study, we investigated whether short-term morphine therapy could alter cardiac autonomic neuropathy and left ventricular dysfunction induced by sepsis in the rat CLP model of sepsis. Pharmacologic and molecular studies were also pursued to determine the role of central neuroinflammatory, oxidative, and chemotactic pathways in the septic–morphine interaction. Experiments were conducted on awake rats pre-equipped with indwelling femoral and intracisternal catheters.

## 2. Results

### 2.1. Hemodynamic and Cardiac Autonomic Effects of CLP

Baseline measurements of hemodynamic and cardiac autonomic activities of sham and CLP rats, measured following hemodynamic stabilization and before drug administration, are presented in Table 1. When compared to sham-operated rats, CLP resulted in significant decrease in mean arterial pressure (MAP) with a value of 15 mmHg (105.5 ± 1.8 vs. 90.5 ± 1.9 mmHg) and significant tachycardia with an approximate value of 44 beats/min (383.2 ± 9.6 vs. 427.2 ± 8.1 beats/min). The fall in MAP was paralleled with significant decrease in time domain (SDNN and rMSSD) and frequency domain parameters (total power and LF/HF) suggesting parasympathomimetic predominance. While the left ventricular contractility index (dP/dt_max_) showed no significant change in CLP compared with sham rats, the isovolumic relaxation constant Tau was significantly decreased by about 60% in septic rats suggesting diastolic dysfunction (Table 1). Raw data of this experiment are provided in Supplementary Files S1–S3.

### 2.2. Morphine Aggravates Septic Manifestations of Hypotension and Autonomic Neuropathy

**Figure 1:**
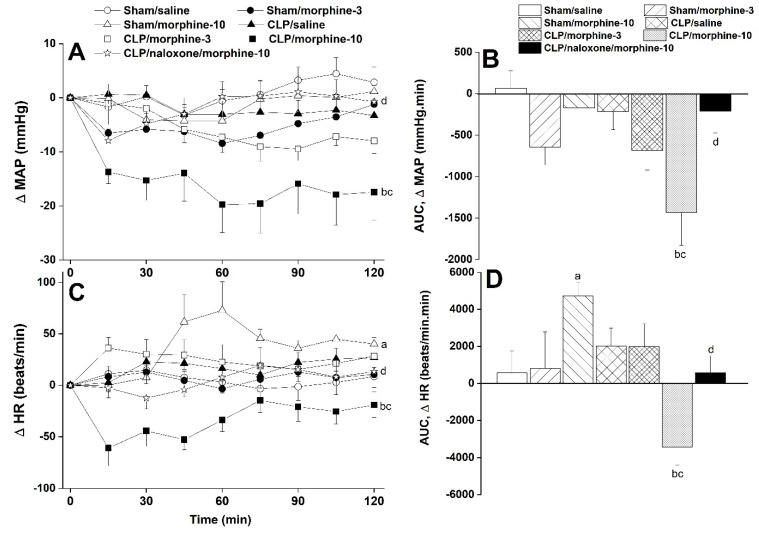
Time-related (**A**–**C**) and cumulative changes (areas under the curves, AUCs, (**B**–**D**)) in mean arterial pressure (MAP) and heart rate (HR) caused by i.v. morphine (3 or 10 mg/kg) in sham-operated and septic (cecal ligation and puncture, CLP) male rats. The effect of opioid receptor antagonism by naloxone (1 mg/kg i.v.) on morphine responses in CLP rats is also shown. Data are presented as means ± SEM from 6–8 observations. Statistical significance was validated using the one-way ANOVA (**A**–**C**) or repeated measures ANOVA (**B**–**D**) followed by the Tukey’s post hoc test. ^a^ *p* < 0.05 vs. “sham/saline”, ^b^ *p* < 0.05 vs. “sham/morphine-10”, ^c^ *p* < 0.05 vs. “CLP/saline”, ^d^ *p* < 0.05 vs. “CLP/morphine-10”.

**Figure 2:**
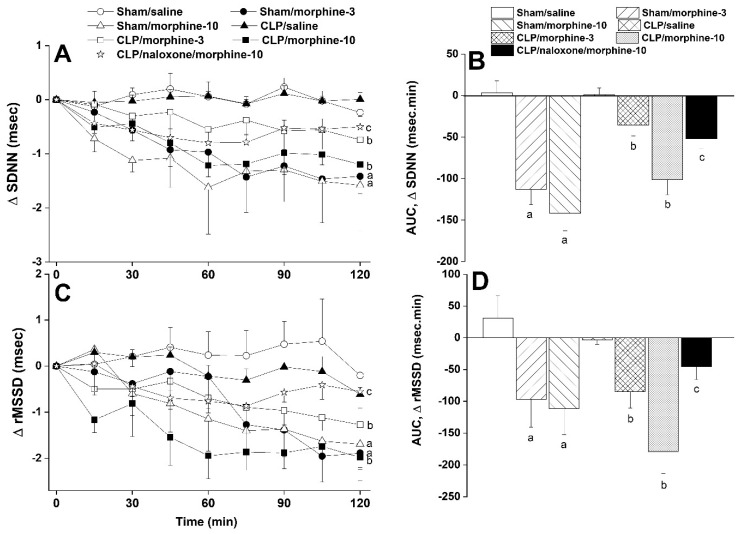
Time-related (**A**–**C**) and cumulative changes (areas under the curve, AUC, (**B**–**D**)) in time-domain indices of HRV (SDNN and rMSSD) evoked by i.v. morphine (3 or 10 mg/kg) in sham-operated and septic (CLP) male rats. The effect of opioid receptor antagonism by naloxone (1 mg/kg i.v.) on morphine responses in CLP rats is also shown. Values are means ± SEM of 6–8 observations. Statistical significance was validated using the one-way ANOVA (**A**–**C**) or repeated measures ANOVA (**B**–**D**) followed by the Tukey’s post hoc test. ^a^ *p* < 0.05 vs. “sham/saline”, ^b^ *p* < 0.05 vs. “CLP/Saline”, ^c^ *p* < 0.05 vs. “CLP/morphine-10”.

**Figure 3:**
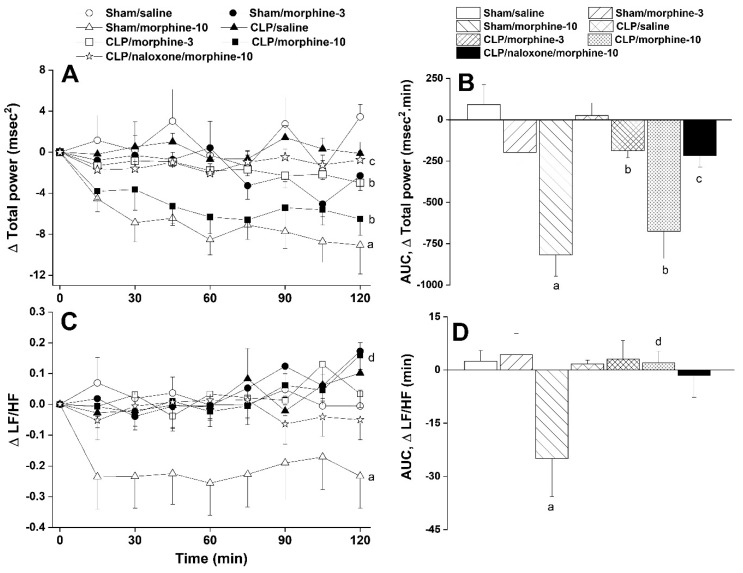
Time-related (**A**–**C**) and cumulative changes (areas under the curve, AUC, (**B**–**D**)) in frequency-domain indices of HRV (total power and LF/HF ratio) induced by i.v. morphine (3 or 10 mg/kg) in sham-operated and septic (cecal ligation and puncture, CLP) male rats. The effect of opioid receptor antagonism by naloxone (1 mg/kg i.v.) on morphine responses in CLP rats is also shown. Values are means ± SEM of 6–8 observations. Statistical significance was validated using the one-way ANOVA (**A**–**C**) or repeated measures ANOVA (**B**–**D**) followed by the Tukey’s post hoc test. ^a^ *p* < 0.05 vs. “sham/saline”, ^b^ *p* < 0.05 vs. “CLP/Saline”, ^c^ *p* < 0.05 vs. “CLP/morphine-10”, ^d^ *p* < 0.05 vs. “sham/morphine-10”.

**Figure 4:**
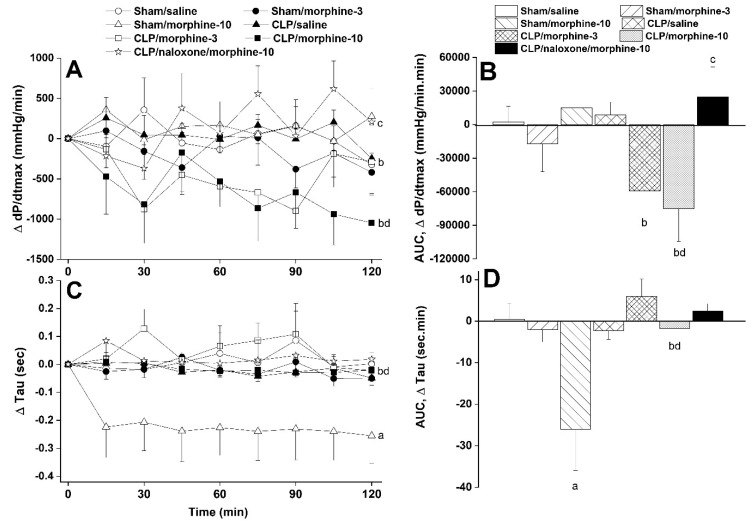
Time-related (**A**–**C**) and cumulative changes (areas under the curve, AUC, (**B**–**D**)) in the maximum rate of rise of blood pressure waves (dP/dtmax) and isovolumic relaxation constant (Tau, a measure of diastolic function) evoked by i.v. morphine (3 or 10 mg/kg) in sham-operated and septic (cecal ligation and puncture, CLP) male rats. The effect of opioid receptor antagonism by naloxone (1 mg/kg i.v.) on morphine responses in CLP rats is also shown. Values are means ± SEM of 6–8 observations. Statistical significance was validated using the one-way ANOVA (**A**–**C**) or repeated measures ANOVA (**B**–**D**) followed by the Tukey’s post hoc test. ^a^ *p* < 0.05 vs. “sham/saline”, ^b^ *p* < 0.05 vs. “CLP/saline”, ^c^ *p* < 0.05 vs. “CLP/morphine-10”, ^d^ *p* < 0.05 vs. “sham/morphine-10”. Supplementary Files S1–S5 contain the raw data illustrating the cardiovascular effects of morphine in CLP rats.

The hemodynamic effects of i.v. administration of morphine (3 and 10 mg/kg) in conscious sham and CLP rats are shown in Figure 1, Figure 2, Figure 3 and Figure 4. In sham rats, the time-related changes in MAP were not significantly affected by either dose of morphine compared with respective saline values (Figure 1A). Similarly, the areas under the curves (AUCs), which represent the cumulative MAP effect of morphine over the entire 2-h experimentation period, resembled those caused by saline (Figure 1B). Alternatively, the treatment of CLP rats with morphine produced dose-related falls in the time-course (Figure 1A) as well as in the cumulative MAP (AUCs, Figure 1B). These morphine effects in CLP rats were significantly greater than respective MAP values seen in saline-treated CLP rats or morphine-treated sham rats. While the HR of sham or CLP rats was not modified by the 3 mg/kg dose of morphine, the 10 mg/kg morphine dose caused opposite changes in HR in sham (increases) and CLP rats (decreases) (Figure 1C,D). Moreover, the prior treatment of CLP rats with the opioid receptor antagonist naloxone (1 mg/kg i.v.) abolished changes in MAP and HR evoked by the higher dose of morphine (Figure 1C,D).

Furthermore, systemic administration of morphine caused significant and dose-related decreases in time (SDNN, Figure 2A,B) and spectral (total power, Figure 3A,B) domain indices of total heart rate variability in both sham and CLP rats. The time-domain maker of cardiac vagotonic activity (rMSSD) was similarly reduced by morphine (Figure 2A,B). The left ventricular contractility index dP/dtmax was significantly reduced by nicotine in CLP rats (Figure 4A,B) whereas the isovolumic relaxation constant Tau remained unaffected (Figure 4C,D). All of the abovementioned morphine effects in CLP rats were significantly abolished by the opioid receptor antagonist naloxone (1 mg/kg, Figure 2, Figure 3 and Figure 4), suggesting a key role for opioid receptors in morphine responses.

### 2.3. Central PI3K/MAPK/NADPHox/Rho Kinase Pathway Mediates the Sepsis/Morphine-Interaction

**Figure 5:**
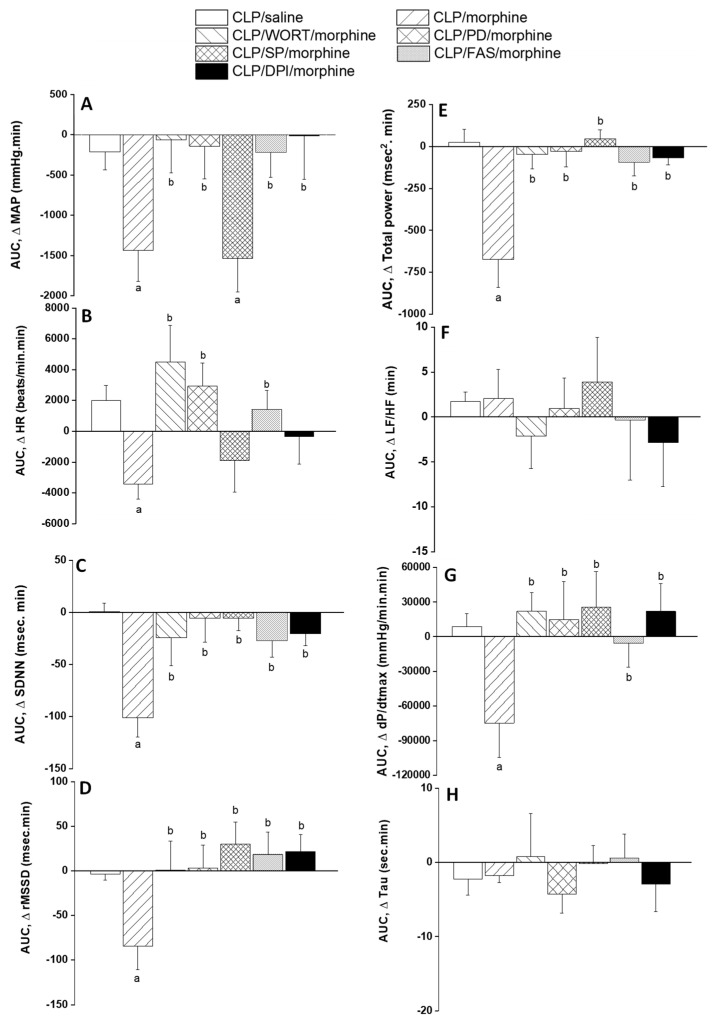
Effects of prior i.c. administration of wortmannin (0.5 μg/5 μL/rat, PI3K inhibitor), PD98056 (10 μg/5 μL/rat, MAPK_ERK1/2_ inhibitor), SP600125 (30 μg/5 μL/rat, MAPK_JNK_ inhibitor), DPI (150 μg/5 μL/rat NADPHox inhibitor) or fasudil (70 μg/5 μL/rat, ROCK inhibitor) on AUCs of the response of mean arterial pressure (MAP, (**A**)), heart rate (HR, (**B**)), time-domain (SDNN, (**C**); rMSSD, (**D**)) and frequency-domain (total power, (**E**); LF/HF ratio, (**F**)) indices of HRV, maximum rate of rise of blood pressure waves (dP/dtmax, (**G**)), and isovolumic relaxation constant (Tau, (**H**)) to morphine (10 mg/kg) in septic rats. Values are means ± SEM of 6–8 observations. The repeated measures ANOVA followed by the Tukey’s post hoc test were employed to test for statistical significance. ^a^ *p* < 0.05 vs. “CLP/Saline”, ^b^ *p* < 0.05 vs. “CLP/morphine”.

This experiment investigated the role of central PI3K/MAPK/NADPHox/ROCK signaling in the cardiovascular effects of morphine in septic rats. AUCs presented in Figure 5A demonstrate that the prior i.c. treatment with the pharmacologic inhibitor of PI3K (wortmannin, 0.5 μg/rat), MAPK-ERK (PD98056, 10 μg/rat), NADPHox (DPI, 150 μg/rat), or ROCK (fasudil, 70 μg/rat) abolished almost completely the hypotensive response to systemically administered morphine (10 mg/kg). By contrast, the BP lowering effect of morphine was preserved following central inhibition of MAPK-JNK by i.c. SP600125 (30 μg/rat). Additionally, only wortmannin, PD98056 and fasudil reversed the bradycardic action of morphine in septic rats (Figure 5B). On the other hand, the morphine-evoked decrements in time (SDNN, Figure 5C) and spectral (total power, Figure 5E) markers of total HRV, cardiac parasympathetic activity (rMSSD, Figure 5D), and cardiac contractility (dP/dtmax, Figure 5G) were indiscriminately blunted by all inhibitors. The cardiac sympatho-vagal balance (LF/HF ratio, Figure 5F) or isovolumic relaxation constant (Tau, Figure 5H) were not altered in any of the treated experimental groups. Supplementary Files S6 and S7 contain the raw data illustrating the effects of i.c. drugs on the cardiovascular morphine/sepsis interaction.

### 2.4. Brainstem Neuroinflammation Provokes Cardiovascular Disturbances Induced by Morphine

**Figure 6:**
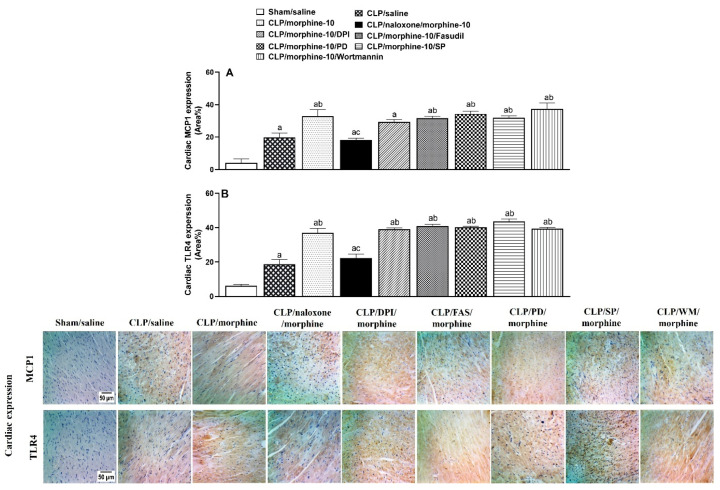
Effects of prior i.v. naloxone (1 mg/kg i.v.) or i.c. administration of wortmannin (0.5 μg/5 μL/rat, PI3K inhibitor), PD98056 (10 μg/5 μL/rat, MAPK_ERK1/2_ inhibitor), SP600125 (30 μg/5 μL/rat, MAPK_JNK_ inhibitor), DPI (150 μg/5 μL/rat NADPHox inhibitor) or fasudil (70 μg/5 μL/rat, ROCK inhibitor) on morphine-10-evoked rises in protein expressions of MCP1 (**A**) and TLR4 (**B**) in hearts of septic rats. Values are means ± SEM of 4–5 observations. ^a^ *p* < 0.05 vs. “sham/saline”, ^b^ *p* < 0.05 vs. “CLP/saline”, ^c^ *p* < 0.05 vs. “CLP/morphine-10”.

**Figure 7:**
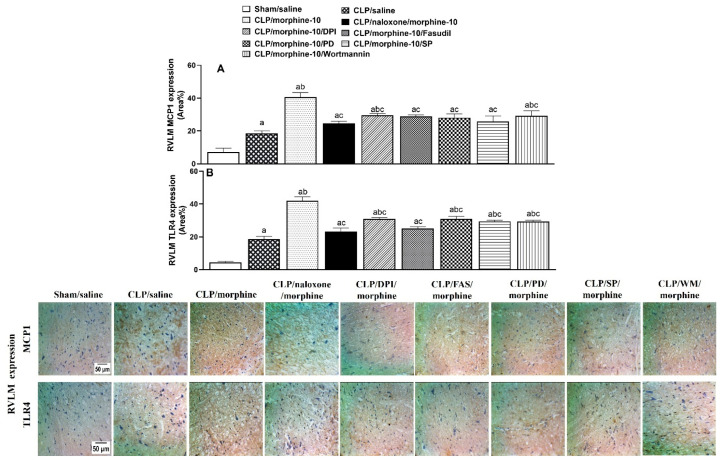
Effects of prior i.v. naloxone (1 mg/kg i.v.) or i.c. administration of wortmannin (0.5 μg/5 μL/rat, PI3K inhibitor), PD98056 (10 μg/5 μL/rat, MAPK_ERK1/2_ inhibitor), SP600125 (30 μg/5 μL/rat, MAPK_JNK_ inhibitor), DPI (150 μg/5 μL/rat NADPHox inhibitor) or fasudil (70 μg/5 μL/rat, ROCK inhibitor) on morphine-10-evoked rises in protein expressions of MCP1 (**A**) and TLR4 (**B**) in the rostral ventrolateral medulla (RVLM) of septic rats. Values are means ± SEM of 5 observations. ^a^ *p* < 0.05 vs. “sham/saline”, ^b^ *p* < 0.05 vs. “CLP/saline”, ^c^ *p* < 0.05 vs. “CLP/morphine-10”. Supplementary Files S8–S10 contain the raw data of the protein expression studies.

Figure 6 and Figure 7 show the effects of pharmacologic blockade of opioid receptors or inhibition of individual components of central PI3K/MAPK/NADPHox/ROCK signaling on the morphine-evoked upregulation of TLR4/MCP1 expression in cardiac and brainstem tissues. The expression of the inflammatory TLR4 and chemotactic MCP1 in heart tissues (Figure 6), as well as in the medullary area of the RVLM (Figure 7), was significantly increased by CLP compared with respective values in control (sham) values. Further increases in TLR4 and MCP1 expression were noted in tissues of morphine (10 mg/kg)-treated CLP rats. The inflammatory and chemotactic actions of morphine in both anatomical locations were reversed by naloxone (1 mg/kg i.v.), suggesting the importance of opioid receptors in mediating the morphine effects (Figure 6 and Figure 7). Moreover, the i.c. administration of the selective inhibitor of PI3K (wortmannin, 0.5 μg/rat), MAPK-ERK (PD98056, 10 μg/rat), MAPK-JNK (SP600125, 30 μg/rat), NADPHox (DPI, 150 μg/rat), or ROCK (fasudil, 70 μg/rat) eliminated the rises evoked by morphine in RVLM, but not cardiac, TLR4/MCP1 expression (Figure 6 and Figure 7). Representative images illustrating the TLR4 and MCP1 staining in cardiac and RVLM sites are shown in Figure 6 and Figure 7, respectively.

## 3. Discussion

The current investigation reveals important insights into the interaction of the prototypic opioid drug morphine with cardiovascular and neuroinflammatory features of sepsis. First, the hypotensive and cardiac autonomic and left ventricular depressant responses elicited by sepsis were accentuated by systemically administered morphine in a dose-related manner. Second, these morphine effects appear to be mediated via the μ-opioid receptor and driven by the intensified inflammatory response to sepsis as suggested by the augmented expression of the inflammatory TLR4 and chemotactic MCP1 signals in peripheral (heart) and brainstem (RVLM) sites. Third, pharmacological studies implicate central inflammatory (PI3K/MAPKs) and oxidative (NADPHox/ROCK) cascades in the deteriorating action of morphine on cardiovascular and neuroinflammatory profiles in septic rats.

The CLP rat model of sepsis was employed in the present study because it closely replicates the features and complexities associated with human sepsis [21]. In accordance with previously reported hallmarks of sepsis [22,23,24], we showed that CLP resulted in significant falls and rises in blood pressure and heart rate. The diastolic function appears to be impaired in septic rats, as indicated by the remarkable decreases in the isovolumic relaxation constant Tau. More importantly, our research revealed that these cardiovascular manifestations of sepsis were variably affected by the simultaneous treatment with morphine. While morphine enhanced the hypotensive response to sepsis, it blunted the associated tachycardia. Further, morphine failed to alter diastolic dysfunction (i.e., reduced Tau) in septic rats, but significantly diminished left ventricular contractility, as suggested by the reduction in the maximal rate of rise of left ventricular pressure (dP/dtmax). Evidently, morphine is believed to negatively modulate cardiovascular functions when used alone. For example, the lowering effect of morphine on blood pressure might be linked to (i) the depression of cardiac sympathetic nerve activity [25], (ii) vasodilation of venous and arterial vasculature due to histamine release [26], or (iii) diminution of cardiac output [27]. This is also consistent with the study by [28], in which morphine provoked the hypovolemic and hypotensive responses induced by acute hemorrhage in sheep through peripheral vasodilation. These effects of morphine may help explain its interaction with the hypotensive and cardiomyopathic effects of sepsis

Experimental and clinical evidence suggests a pivotal role for autonomic imbalances in the cardiovascular insults caused by sepsis and septic shock. The normalization of cardiac autonomic activity is an important therapeutic goal alongside conventional resuscitation maneuvers in the management of sepsis complications [29,30]. In the present investigation, the assessment of HRV in the time and frequency domains was employed to determine the role of cardiac autonomic activity in the sepsis/morphine interaction. In line with previous studies [31,32], cardiac autonomic dysfunction in the current sepsis model was corroborated by the significant reductions in time- and frequency-domain indices of total HRV, SDNN and total power, respectively, compared with sham-operated rats (Table 1). Further, considering that the spectral bands of LF and HF mark cardiac sympathetic and parasympathetic activities, respectively [33,34], the reduction in LF/HF ratio of the spectral profile is consistent with the shift in cardiac autonomic balance towards parasympathetic dominance. Like its adverse effect on the hypotensive and left ventricular depressant effects of sepsis, the cardiac parasympathetic dominance seen in septic rats was maintained following morphine treatment while the depressed time- and spectral indices of total HRV showed further decreases. Given that the adverse cardiovascular effects of morphine in our study were eliminated or at least attenuated by prior administration of the µ-opioid receptor antagonist naloxone, the presence of functional µ-opioid receptors appears to be necessary for the effects of morphine to be manifested.

Central PI3K/MAPKs signaling has been implicated in individual insults caused by sepsis or morphine. Indeed, the mutual facilitatory interaction between PI3K and MAPK entities are key components of the hyperinflammatory phase of sepsis triggered by TLR4 [35], the sensing receptor for microbial structures such as bacterial lipopolysaccharides and associated molecular patterns [36]. Moreover, the activation of TLR4-dependent PI3K/MAPKs promotes nuclear translocation of NFκB, and a consequent surge in proinflammatory signals such as TNFα and interleukins [37]. Alternatively, a role for of PI3K/MAPK pathway in the biological action of morphine such as hyperalgesia [38], angiogenesis [39], and neurotoxicity [40] has been documented. This prompted us to investigate whether central PI3K/MAPKs could be blamed for the amplifying action of morphine on cardiovascular sequels of sepsis. The data showed that all cardiovascular effects of morphine in septic rats disappeared following central inhibition of PI3K or MAPK-ERK by i.c. wortmannin and PD98056, respectively. In contrast, the inhibition of central MAPK-JNK by SP600125 abolished the depressant effects of morphine on cardiac autonomic activity and left ventricular contractility without modifying the associated hypotension or tachycardia. These results suggest uneven roles of the two MAPK isoforms (ERK and JNK) in mediating the actions of morphine. This assumption in confirmed by reports that MAPK isoforms influence morphine analgesia through distinct modulatory pathways [38,41]

**Figure 8:**
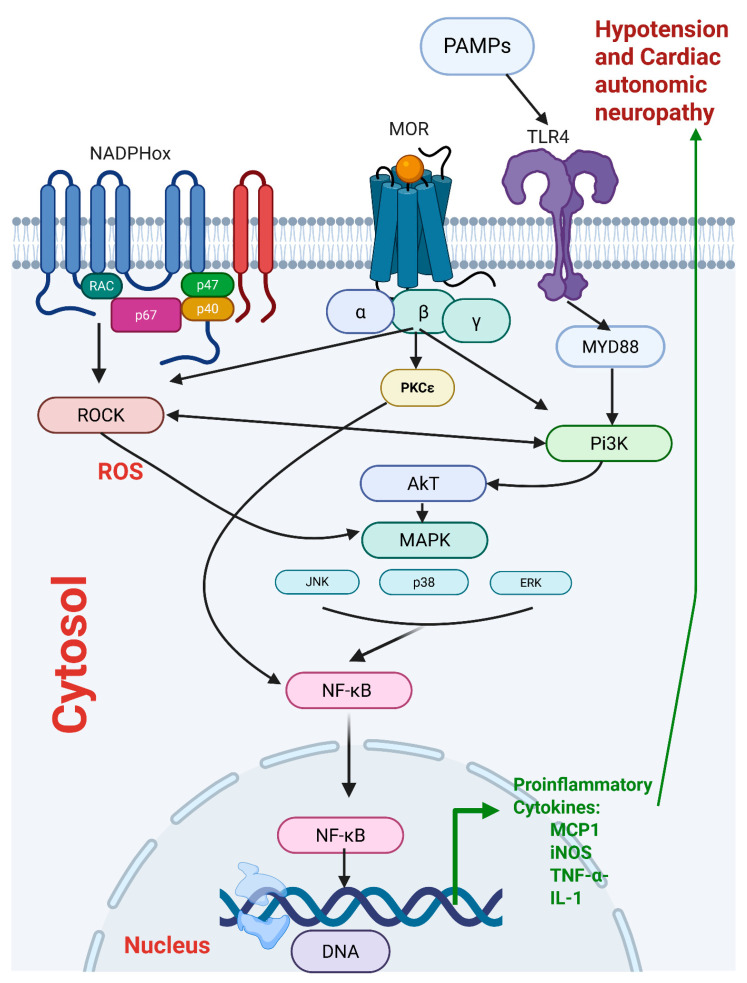
Schematic presentation of possible central signaling pathways involved in the interaction between μ-opioid receptors and inflammatory and oxidative pathways in sepsis. PAMPS: pathogen-associated molecular patterns, TLR4: Toll-Like Receptor-4, MYD88: Myeloid Differentiation primary response 88, NADPHox: Nicotinamide adenine dinucleotide phosphate oxidase, MOR: μ-opioid receptor, Pi3K: Phosphoinositide-3 kinases, AkT: Protein kinase B, PKC: Protein kinase C epsilon type, ROCK: Rho-associated coiled-coil kinase, ROS: Reactive Oxygen Species, MAPK: Mitogen-activated protein kinase, JNK: c-Jun N-terminal Kinase, ERK: Extracellular signal-regulated kinase, NF-κB: Nuclear Factor kappa-B, MCP1: Monocyte Chemoattractant Protein-1, iNOS: Inducible Nitric Oxide Synthase, TNF- α: Tumor Necrosis Factor-alpha, IL-1: Interlukin-1.

Inflammation and oxidative stress are mutually facilitatory cellular processes that magnify tissue damage and disease pathogenesis [42,43]. Convincing evidence exists that reactive oxygen species generated through the NADPHox/ROCK pathway play a crucial role in amplifying the inflammatory storm triggered by sepsis and other inflammatory disorders [44,45]. Reactive oxygen radicals serve to activate PI3K and downstream effectors, like the transcription factor NF-κB, which regulates innate and adaptive immune functions via encoding a large number of inflammatory cytokines and chemokines [46]. Likewise, morphine has been shown to activate NADPHox/ROCK signaling and increase superoxide generation [47,48]. Activation of μ-opioid receptors also upregulates NF-κB in LPS models [49], whereas the blocking of these receptors reduces neuroinflammation [50]. These findings are echoed by microinjection studies of the present study, in which the central administration of diphenyleneiodonium (NADPHox inhibitor) and fasudil (ROCK inhibitor) into the cisterna magnum blunted hemodynamic, autonomic, and left ventricular deficits caused by morphine in the setting of sepsis. Together, the data highlight a major role of the interconnected inflammatory PI3K/MAPK and oxidative NADPHox/ROCK pathways in the exacerbating action of morphine on cardiovascular manifestations of sepsis. A hypothesized schematic diagram of the mutual interaction between these pathways and μ-opioid receptors is depicted in Figure 8.

To reinforce the role of the PI3K/MAPK/NADPHox/ROCK cascade in morphine responses, we measured the immunohistochemical protein expression of TLR4 and MCP1 in the heart, as well as in neuronal pools of brainstem RVLM. While TLR4 triggers the inflammatory response after dimerization with microbial endotoxins [36], MCP1 is a vital chemokine that regulates the migration and infiltration of inflammatory cells, such as monocytes and macrophages [51]. Our finding that morphine augmented the sepsis-evoked rises in cardiac/RVLM expression of TLR4 and MCP1 indicate that peripheral and central pathways of these signaling molecules contribute the deteriorated cardiovascular profile of morphine-treated septic rats. Additionally, the exaggerated expression of TLR4 and MCP1 in this model system was significantly attenuated in RVLM, but not in the heart, after pharmacologic inhibition of individual components of PI3K/MAPK NADPHox/ROCK. The lack of effect of the inhibitors in the heart is expected considering that all inhibitors were centrally administered into the cisterna magnum. Importantly, this does not rule out the involvement of peripherally mediated molecular targets, such as μ-opioid receptors, in the interaction between morphine and sepsis. This is supported by our observation that systemically administered naloxone significantly reduced TLR4 and MCP1 expression in both cardiac and RVLM tissues. It should be mentioned that we specifically targeted the RVLM because this brainstem neuroanatomical area has been shown to play a fundamental role in central mediation of cardiovascular control [52] and in central processing of peripheral inflammatory stimuli associated with sepsis [53].

It is important to comment on three possible limitations of this study. First, fasudil, wortmannin, DPI, SP600125 and PD98056 were administered centrally to inhibit specific inflammatory and oxidative signals, but the contribution of other potential off-target sites to the observed effects of these inhibitors cannot be overlooked. For instance, in addition to ROCK inhibition, fasudil can also inhibit protein kinases A and G, especially at higher doses [54]. The PI3K inhibitor wortmannin was also found to inhibit other PI3K-related kinases, such as DNA-dependent protein kinase and ataxia telangiectasia mutated kinase [55]. Further, DPI inhibits NADPHox and other flavoprotein containing targets, such as nitric oxide synthases and xanthine oxidase [56]. Second, the use of the intracisternal route for the administration of individual pharmacologic inhibitors of the PI3K/MAPK/NADPHox/ROCK signaling raises doubts regarding the clinical relevance of the present data. Despite its importance in understanding central neuronal circuits involved in the pathogenesis of diseases and design and development of pharmacotherapies, central drug administration is not feasible in humans except, probably, in rare cases such as intrathecal baclofen for spasticity control [57]. Of note, the neuroprotective effects of these inhibitors after systemic administration have been validated [58,59,60,61,62,63]. Third, since our study was performed in male rats, it is not clear whether a similar interaction would be observed in females. Given the accumulated evidence from previous clinical and experimental studies including our own regarding a sex bias in integrated and molecular cardiovascular consequences of sepsis [64,65], further research is needed to explore the interaction between morphine and sepsis in females and to elucidate the role of gonadal hormones in this context.

In conclusion, this study demonstrates that cardiovascular markers of sepsis, such as hypotension, cardiac autonomic neuropathy, and myocardial depression, are enhanced following concurrent exposure to morphine. Pharmacologic and protein expression studies implicate central inflammatory TLR4 and downstream PI3K/MAPK/NADPHox/ROCK signaling in the interaction. Clinically, the pharmacologic inhibition of multiple molecules along this signaling cascade could be exploited as a potential therapeutic strategy to minimize cardiovascular consequences that may arise from opioid use in septic intensive care unit patients.

### Perspectives

Sepsis is globally recognized as a systemic inflammatory response to infection and a leading cause of morbidity and mortality in intensive care units. While opioids are the cornerstone of pain management in critical care, our experimental observations that morphine, a prototypical opioid, exacerbates neuroinflammatory, hemodynamic and cardiac autonomic neuropathic effects of sepsis raise serious concerns about the potential for increased cardiovascular risk in this setting. If these preclinical observations are translated to humans, the clinical use of morphine in patients with sepsis, or perhaps in similar states of cardiovascular dysfunction or autonomic instability, may need to be reconsidered.

## 4. Materials and Method

### 4.1. Animals

Adult male Wistar rats (200–250 g) were used (Faculty of Pharmacy animal facility, Alexandria University, Alexandria, Egypt). Rats were kept at an ambient temperature and had a free access to standard rat chow with 19% protein and water. A total of 96 rats were used in this study. The sample size calculation was performed based on power analysis using G*Power 3.1.9.7 software [66]. All animal protocols were approved by the Institutional Animal Care and Use Committee of Alexandria University, Egypt (Approval No. AU/06.2020.6.7.2.73) and complied with the ARRIVE guidelines (https://arriveguidelines.org/, accessed on 12 February 2021). The ARRIVE-Author Checklist is attached (see Supplementary File S11).

### 4.2. Drugs

Morphine (Masr Pharmaceutical Co., Cairo, Egypt), Heparin Sodium (Heparin^®^ ampoules 5000 IU/mL, Nile Pharmaceutical Co., Cairo, Egypt), Thiopental (Thiopental^®^, Biochemie GmbH, Vienna, Austria), Naloxone (SERB pharmaceutical Co., Paris, France), diphenyleneiodonium (DPI); fasudil (Tocris Bioscience, Bristol, UK), wortmannin, PD98056 and SP600125 (Sigma-Aldrich, St. Louis, MO, USA). Morphine, naloxone, and heparin were provided as injectable liquid solutions and diluted with saline as appropriate. All other drugs (wortmannin, PD98056, SP600125, fasudil and DPI) were received as powders and dissolved in DMSO.

### 4.3. Cecal Ligation and Puncture (CLP)

CLP was performed as described in our previous studies [67] and by others [68]. The abdominal region of rats anesthetized with thiopental (50 mg/kg, i.p.) was shaved and disinfected using a betadine solution. A 1-cm midline laparotomy was performed, and the cecum was exposed. The distal one third end of the cecum was ligated and punctured three times on the same side using a 21-guage needle and then gently compressed to expel a small amount of fecal content. The cecum was then returned to the abdominal cavity, and the abdominal musculature and skin were stitched.

### 4.4. Intracisternal Cannulation (i.c.)

Four days before cardiovascular measurements (i.e., 3 days before intravascular catheterization and CLP), a stainless steel guide cannula (23 G) was implanted into the cisterna magna of thiopental -anesthetized rats (50 mg/kg, i.p.) [69,70]. The guide cannula was inserted between the occipital bone and the cerebellum, with its tip positioned in the cisterna magna, and was then fixed in place using dental acrylic cement. The guide cannula was deemed patent upon observation of spontaneous cerebrospinal fluid outflow. Following i.c. cannulation, rats were housed individually. 

### 4.5. Intravascular Cannulation

The detailed method was described in our previous studies [71,72]. Immediately after CLP or sham operation, catheters (each consisting of 5 cm polyethylene-10 tubing bonded to 15 cm polyethylene-50 tubing) were placed in the abdominal aorta and vena cava via the femoral artery and vein, respectively, for blood pressure measurement and intravenous drug administration. The polyethylene-10 portion was used for the intravascular segment of the catheter. The catheters were tunneled subcutaneously and exteriorized at the back of the neck between the scapulae. They were then flushed with heparin (0.2 mL; 100 U/mL) and plugged by stainless steel pins. On the following day, the arterial catheter was connected to a BP transducer (model P23XL; Astro-Med, West Warwick, RI, USA), which was connected through MLAC11 Grass adapter cable to a computerized data acquisition system with LabChart-7 pro software (Power Lab 4/35, model ML866/P; AD Instruments Pty Ltd., Castle Hill, Australia) for BP, heart rate (HR) and HRV assessment, as described later.

### 4.6. Time-Domain Analysis of HRV

As described elsewhere [33,73], two time-domain parameters of cardiac autonomic function were assessed. First, the standard deviation of the R–R interval (SDNN), which measures the overall cardiac autonomic activity and correlates with the total power of the frequency domain. The R–R intervals were computed as the reciprocal of HR in milliseconds. The second time-domain parameter was the square root of the mean squared differences of successive R–R intervals (rMSSD), a measure of cardiac parasympathetic function, and relates to the high frequency (HF) power of the frequency spectrum. SDNN and rMSSD were measured before (baseline) and at 15 min intervals after drug treatments.

### 4.7. Frequency-Domain Analysis of HRV

This method employs the Fast Fourier Transform (FFT) algorithm to analyze the power spectrum density of the R–R interval data, where the HRV signal is broken into different frequency components that provide important information about cardiac autonomic activity. The data were interpolated to generate equally spaced samples at an effective sampling frequency of 10 Hz. A second-order interpolation method was used to create a smooth curve through the existing data points, providing a smoother visual representation. Equidistant sampling enabled direct spectral analysis using FFT algorithm. The resulting spectra were divided into two specific frequency bands, LF (0.25–0.75 Hz) and HF (0.75–3 Hz). Total spectral power was used as an index of overall cardiac autonomic activity, while the LF/HF ratio served as an indicator of sympatho-vagal balance. Spectral parameters were evaluated at baseline and in 15-min intervals following drug administration.

### 4.8. Immunohistochemistry

The expression of TLR-4 and MCP1 in the left ventricle and brainstem RVLM was measured as described in previous studies. Tissues were fixed in 10% formalin solution overnight, dehydrated in a graded series of ethanol (70, 95 and 100%), then embedded in paraffin. Tissue sections of 5 µm thickness of the rat heart and RVLM (−12.0 mm to −12.48 mm relative to bregma [74]) were placed on positively charged adhesion microscope slides, deparaffinized in xylene and rehydrated through a graded ethanol series (100%, 95%, and 70%). The slides were gently rinsed with PBS and allowed to drain. Heat-induced epitope retrieval was performed by immersing the slides in Coplin jars containing 10 mM citrate buffer and heating them in a microwave, first at full power (100%) for 1 min, followed by reduced power (30%) for 9 min. After retrieval, the sections were rinsed with 1× TBST buffer (50 mM Tris-HCl, pH 7.4, 150 mM NaCl, 0.1% Tween 20). Endogenous peroxidase activity was quenched using 3% hydrogen peroxide, followed by washing with 1× TBST. A universal protein block was then applied for 20 min. The appropriate primary monoclonal antibodies (rabbit anti-TLR4 and rabbit anti-MCP1), diluted 1:300 according to the manufacturer’s instructions, were applied to the slides, which were then incubated overnight at 4 °C. After incubation, the slides were washed with 1× TBST, rinsed, and incubated for 30 min with the HRP-conjugated secondary antibody. The chromogen 3,3′-diaminobenzidine (DAB) was prepared and applied according to the manufacturer’s instructions to visualize protein expression. Slides were counterstained with hematoxylin, then passed through increasing concentrations of alcohol followed by xylene. Images were captured using an Optikam B9 digital camera (Optika^®^ Microscopes, Ponteranica, Italy), and immunohistochemical signals in the heart and brainstem RVLM were quantified using Fiji ImageJ software (version 1.51n, National Institutes of Health, Bethesda, MD, USA) with the color deconvolution plugin. This plugin splits the stained image into three separate color channels: hematoxylin (blue, color 1), DAB (brown, color 2) and background (color 3). The intensity of the brown DAB color was then measured as the percentage of the area above a cut-off threshold.

### 4.9. Protocols and Experimental Design

**Figure 9:**
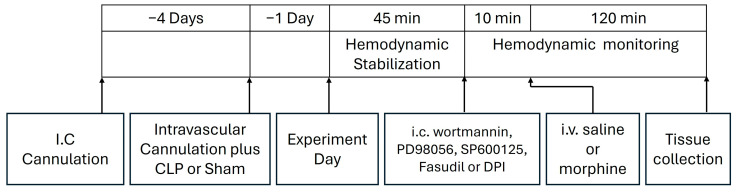
Diagrammatic representation of the timeline of surgical procedures and drug regimens.

This experiment tested the dose-related effect of systemically administered morphine on cardiovascular and inflammatory manifestations of sepsis. Male Wistar rats were randomly assigned to one of the following 7 experimental groups (*n* = 6–8 each): (i) sham/saline, (ii) sham/morphine (3 mg/kg), (iii) sham/morphine (10 mg/kg), (iv) CLP/saline, (v) CLP/morphine (3 mg/kg), (vi) CLP/morphine (10 mg/kg), (vii) CLP/naloxone (1 mg/mL)/morphine (10 mg/kg). Following a stabilization period of at least 45 min, saline or morphine was administered intravenously, and hemodynamic parameters were monitored for an additional 2 h. The doses of morphine employed in this investigation have been repeatedly used in previous studies [75,76,77,78]. Changes in MAP, HR, and HRV indices, both time-domain (SDNN, rMSSD) and frequency-domain (total power, LF 0.25–0.75 Hz; HF 0.75–3 Hz, LF/HF ratio), were assessed at 15-min intervals. Left ventricular function was evaluated by calculating the following: (i) the isovolumic relaxation time constant (Tau), reflecting the exponential decline in ventricular pressure during isovolumic relaxation, and (ii) the maximal rate of pressure rise (dP/dt_max), an indicator of left ventricular contractility [79]. At the conclusion of hemodynamic monitoring, rats were euthanized with an overdose of thiopental (100 mg/kg). The heart and brainstem were dissected, fixed in 10% formaldehyde, and processed for immunohistochemical analysis of TLR-4 and MCP1 protein expression, as previously described. A schematic overview of the surgical procedures and drug administration timelines is shown in Figure 9. Blinding was not feasible, as the same researcher performed both the surgical interventions and drug treatments.

The roles of the inflammatory PI3K/MAPK/NADPHox/ROCK pathways in the septic-morphine interaction were also investigated. Five more CLP rat groups (*n* = 6–8 each) were employed and randomly assigned to receive one of the following regimens: (i) i.c. wortmannin (0.5 μg/5 μL/rat, PI3K inhibitor) + morphine (10 mg/kg i.v.), (ii) i.c. PD98056 (10 μg/5 μL/rat, MAPK_ERK1/2_ inhibitor) + morphine (10 mg/kg i.v.), (iii) i.c. SP600125 (30 μg/5 μL/rat, MAPK_JNK_ inhibitor) + morphine (10 mg/kg i.v.), (iv) i.c. fasudil (70 μg/5 μL/rat, ROCK inhibitor) + morphine (10 mg/kg i.v.), (v) i.c. DPI (150 μg/5 μL/rat NADPHox inhibitor)+ morphine (10 mg/kg i.v.). These drugs were dissolved in DMSO and their doses were selected based on our reported studies and others [80,81,82]. The sole effect of DMSO was not studied in our current study, but our previous studies [80] and others [83] demonstrated no significant hemodynamic changes following i.c. administration of DMSO. A 10-min interval was maintained between each treatment within the regimen, and hemodynamic monitoring continued for 2 h following the final treatment. MAP, HR, HRV, and left ventricular function were assessed at 15-min intervals. After the monitoring period, rats were euthanized with an overdose of thiopental (100 mg/kg), and the hearts and brainstems were collected and processed for immunohistochemical analysis of TLR-4 and MCP1 protein expression, as previously described. Figure 9 presents a schematic timeline of the surgical procedures and drug administration protocols used in the study.

### 4.10. Statistical Analysis

Data are presented as means ± S.E.M. The area under the curve (AUC) for each parameter was calculated to assess the cumulative drug effect over the duration of the experiment. AUCs were determined using GraphPad Prism version 8.0.2, applying trapezoidal integration with the zero-line set as the baseline. The analysis accounted for both positive and negative deviations from baseline, and the net AUC was obtained by subtracting the area of values below the baseline from those above it [71]. Statistical comparisons were made using one-way or repeated measures ANOVA, followed by Tukey’s post hoc test for multiple comparisons. All statistical analyses were conducted using GraphPad InStat version 3.05. A *p*-value of less than 0.05 was considered statistically significant.

## Figures and Tables

**Table 1:** Baseline values measured 24 h after sham operation or CLP.

Parameter	Sham	CLP
MAP, mmHg	105.5 ± 1.8	90.5 ± 1.9 *
HR, beats/min	383.2 ± 9.6	427.2 ± 8.1 *
SDNN, msec	3.98 ± 0.21	2.59 ± 0.11 *
rMSSD, msec	4.23 ± 0.31	2.99 ± 0.21 *
Total power, msec^2^	14.22 ± 1.54	6.33 ± 0.48 *
LF/HF	0.23 ± 0.03	0.12 ± 0.02 *
+dP/dtmax, mmHg/min	2998.0 ± 256.6	3242.6 ± 245.7
Tau, sec	0.246 ± 0.06	0.094 ± 0.010 *

Values are means ± SEM of 6–8 observations. * *p* < 0.05 vs. corresponding sham values. MAP, mean arterial pressure; HR, heart rate; SDNN, standard deviation of NN intervals; rMSSD, square root of the mean squared differences of successive NN intervals; LF/HF, low frequency/high frequency ratio; +dP/dtmax, maximal rate of rise of left ventricular pressure; Tau, isovolumic relaxation constant.

## Data Availability

Raw data have been uploaded as Supplementary Materials.

## Supplementary Materials

The following supporting information can be downloaded at: https://www.mdpi.com/article/10.3390/ph18060882/s1, File S1: CLP-Morphine, MAP-HR; File S2: CLP-Morphine, HRV-LVF; File S3: Pooled Baselines for Sham- CLP; File S4: Naloxone, BP HR; File S5: CLP-Naloxone-Morph, HRV-LVF; File S6: IC drugs BP-HR; File S7: IC drugs HRV; File S8: CLP-Morph-IC drugs, TLR4; File S9: CLP-Morph-IC drugs, MCP1; File S10: Uncropped Photos for cardiac immunohistochemical expression; File S11: ARRIVE-Author Checklist.

## Abbreviations

The following abbreviations are used in this manuscript:

AUCs	Areas Under the Curves
CLP	Cecal ligation and puncture
dP/dtmax	maximal rate of rise of left ventricular pressure
ERK	Extracellular signal-regulated kinase
HR	Heart Rate
HRV	Heart Rate Variability
i.c.	Intracisternal cannulation
ICUs	Intensive Care Units
JNK	c-Jun N-terminal Kinase
LF/HF	low frequency/high frequency ratio
MAPK	Mitogen-activated protein kinase
MAP	Mean Arterial Pressure
MCP1	Monocyte Chemoattractant Protein-1
NADPHox	Nicotinamide adenine dinucleotide phosphate oxidase
PI3K	Phosphoinositide-3 kinases
rMSSD	the square root of the mean squared differences of successive NN intervals
ROCK	Rho-associated coiled-coil kinase
RVLM	Rostral Ventrolateral Medullary
SDNN	Standard Deviation of NN intervals
TLR4	Toll-Like Receptor 4
TNF-α	Tumor Necrosis Factor-alpha

## References

[1] Singer M., Deutschman C.S., Seymour C.W., Shankar-Hari M., Annane D., Bauer M., Bellomo R., Bernard G.R., Chiche J.D., Coopersmith C.M. (2016). The Third International Consensus Definitions for Sepsis and Septic Shock (Sepsis-3). JAMA.

[2] Deutschman C.S., Tracey K.J. (2014). Sepsis: Current dogma and new perspectives. Immunity.

[3] Moraes C.A., Zaverucha-do-Valle C., Fleurance R., Sharshar T., Bozza F.A., d’Avila J.C. (2021). Neuroinflammation in Sepsis: Molecular Pathways of Microglia Activation. Pharmaceuticals.

[4] Sallam M.Y., El-Gowilly S.M., Abdel-Galil A.G., El-Mas M.M. (2016). Central GABAA receptors are involved in inflammatory and cardiovascular consequences of endotoxemia in conscious rats. Naunyn-Schmiedeberg’s Arch. Pharmacol..

[5] Sayk F., Vietheer A., Schaaf B., Wellhoener P., Weitz G., Lehnert H., Dodt C. (2008). Endotoxemia causes central downregulation of sympathetic vasomotor tone in healthy humans. Am. J. Physiol.-Regul. Integr. Comp. Physiol..

[6] Filiz A.I., Ozturk A., Kurt Y., Sucullu I., Akin M.L., Yildiz M. (2010). The effects of immunosuppressive agents on inflammatory response in septic rats. Cent. Eur. J. Med..

[7] Zhu T., Liao X., Feng T., Wu Q., Zhang J., Cao X., Li H. (2017). Plasma Monocyte Chemoattractant Protein 1 as a Predictive Marker for Sepsis Prognosis: A Prospective Cohort Study. Tohoku J. Exp. Med..

[8] Tunctan B., Korkmaz B., Cuez T., Kemal Buharalioglu C., Sahan-Firat S., Falck J., Malik K.U. (2010). Contribution of vasoactive eicosanoids and nitric oxide production to the effect of selective cyclooxygenase-2 inhibitor, NS-398, on endotoxin-induced hypotension in rats. Basic Clin. Pharmacol. Toxicol..

[9] Temiz-Resitoglu M., Kucukkavruk S.P., Guden D.S., Cecen P., Sari A.N., Tunctan B., Gorur A., Tamer-Gumus L., Buharalioglu C.K., Malik K.U. (2017). Activation of mTOR/IκB-α/NF-κB pathway contributes to LPS-induced hypotension and inflammation in rats. Eur. J. Pharmacol..

[10] Anderberg S.B., Luther T., Frithiof R. (2017). Physiological aspects of Toll-like receptor 4 activation in sepsis-induced acute kidney injury. Acta Physiol..

[11] Cazareth J., Guyon A., Heurteaux C., Chabry J., Petit-Paitel A. (2014). Molecular and cellular neuroinflammatory status of mouse brain after systemic lipopolysaccharide challenge: Importance of CCR2/CCL2 signaling. J. Neuroinflamm..

[12] Wennström M., Janelidze S., Bay-Richter C., Minthon L., Brundin L. (2014). Pro-Inflammatory Cytokines Reduce the Proliferation of NG2 Cells and Increase Shedding of NG2 In Vivo and In Vitro. PLoS ONE.

[13] Chaudhry H., Zhou J., Zhong Y., Ali M.M., McGuire F., Nagarkatti P.S., Nagarkatti M. (2013). Role of cytokines as a double-edged sword in sepsis. In Vivo.

[14] Sessler C.N., Wilhelm W. (2008). Analgesia and sedation in the intensive care unit: An overview of the issues. Crit. Care.

[15] Zhang R., Meng J., Lian Q., Chen X., Bauman B., Chu H., Segura B., Roy S. (2018). Prescription opioids are associated with higher mortality in patients diagnosed with sepsis: A retrospective cohort study using electronic health records. PLoS ONE.

[16] Hu A.M., Shan Z.M., Zhang Z.J., Li H.P. (2021). Comparative Efficacy of Fentanyl and Morphine in Patients with or At Risk for Acute Respiratory Distress Syndrome: A Propensity Score-Matched Cohort Study. Drugs R D.

[17] Nardi G.M., Bet A.C., Sordi R., Fernandes D., Assreuy J. (2013). Opioid analgesics in experimental sepsis: Effects on physiological, biochemical, and haemodynamic parameters. Fundam. Clin. Pharmacol..

[18] Meng J., Banerjee S., Li D., Sindberg G.M., Wang F., Ma J., Roy S. (2015). Opioid Exacerbation of Gram-positive sepsis, induced by Gut Microbial Modulation, is Rescued by IL-17A Neutralization. Sci. Rep..

[19] Banerjee S., Meng J., Das S., Krishnan A., Haworth J., Charboneau R., Zeng Y., Ramakrishnan S., Roy S. (2013). Morphine induced exacerbation of sepsis is mediated by tempering endotoxin tolerance through modulation of miR-146a. Sci. Rep..

[20] Brunauer A., Koköfer A., Bataar O., Gradwohl-Matis I., Dankl D., Dünser M.W. (2014). The arterial blood pressure associated with terminal cardiovascular collapse in critically ill patients: A retrospective cohort study. Crit. Care.

[21] Dejager L., Pinheiro I., Dejonckheere E., Libert C. (2011). Cecal ligation and puncture: The gold standard model for polymicrobial sepsis?. Trends Microbiol..

[22] Rudiger A., Singer M. (2013). The Heart in Sepsis: From Basic Mechanisms to Clinical Management. Curr. Vasc. Pharmacol..

[23] Hoover D.B., Ozment T.R., Wondergem R., Li C., Williams D.L. (2015). Impaired heart rate regulation and depression of cardiac chronotropic and dromotropic function in polymicrobial sepsis. Shock.

[24] Pancoto J.A., Correa P.B., Oliveira-Pelegrin G.R., Rocha M.J. (2008). Autonomic dysfunction in experimental sepsis induced by cecal ligation and puncture. Auton. Neurosci..

[25] Ellingsrud C., Agewall S. (2016). Morphine in the treatment of acute pulmonary oedema—Why?. Int. J. Cardiol..

[26] Afshari R., Maxwell S.R., Webb D.J., Bateman D.N. (2009). Morphine is an arteriolar vasodilator in man. Br. J. Clin. Pharmacol..

[27] Chen A., Ashburn M.A. (2015). Cardiac Effects of Opioid Therapy. Pain Med..

[28] Frithiof R., Rundgren M. (2006). Activation of central opioid receptors determines the timing of hypotension during acute hemorrhage-induced hypovolemia in conscious sheep. Am. J. Physiol. Regul. Integr. Comp. Physiol..

[29] Carrara M., Ferrario M., Bollen Pinto B., Herpain A. (2021). The autonomic nervous system in septic shock and its role as a future therapeutic target: A narrative review. Ann. Intensive Care.

[30] Adam J., Rupprecht S., Künstler E.C.S., Hoyer D. (2023). Heart rate variability as a marker and predictor of inflammation, nosocomial infection, and sepsis—A systematic review. Auton. Neurosci..

[31] Barnaby D.P., Fernando S.M., Ferrick K.J., Herry C.L., Seely A.J.E., Bijur P.E., Gallagher E.J. (2018). Use of the low-frequency/high-frequency ratio of heart rate variability to predict short-term deterioration in emergency department patients with sepsis. Emerg. Med. J..

[32] de Castilho F.M., Ribeiro A.L.P., Nobre V., Barros G., de Sousa M.R. (2018). Heart rate variability as predictor of mortality in sepsis: A systematic review. PLoS ONE.

[33] Stein P.K., Bosner M.S., Kleiger R.E., Conger B.M. (1994). Heart rate variability: A measure of cardiac autonomic tone. Am. Heart J..

[34] El-Mas M.M., Abdel-Rahman A.A. (2007). Intermittent clonidine regimen abolishes tolerance to its antihypertensive effect: A spectral study. J. Cardiovasc. Pharmacol..

[35] Laird M.H., Rhee S.H., Perkins D.J., Medvedev A.E., Piao W., Fenton M.J., Vogel S.N. (2009). TLR4/MyD88/PI3K interactions regulate TLR4 signaling. J. Leucoc. Biol..

[36] Pandey S., Kawai T., Akira S. (2014). Microbial sensing by Toll-like receptors and intracellular nucleic acid sensors. Cold Spring Harb. Perspect. Biol..

[37] Zhao X., Wang M., Zhang Y., Zhang Y., Tang H., Yue H., Zhang L., Song D. (2024). Macrophages in the inflammatory response to endotoxic shock. Immun. Inflamm. Dis..

[38] de Freitas B.G., Pereira L.M., Santa-Cecília F.V., Hösch N.G., Picolo G., Cury Y., Zambelli V.O. (2019). Mitogen-Activated Protein Kinase Signaling Mediates Morphine Induced-Delayed Hyperalgesia. Front. Neurosci..

[39] Wang Z., Jiang L., Wang J., Chai Z., Xiong W. (2021). Morphine promotes angiogenesis by activating PI3K/Akt/HIF-1α pathway and upregulating VEGF in hepatocellular carcinoma. J. Gastrointest. Oncol..

[40] Malik S., Khalique H., Buch S., Seth P. (2011). A Growth Factor Attenuates HIV-1 Tat and Morphine Induced Damage to Human Neurons: Implication in HIV/AIDS-Drug Abuse Cases. PLoS ONE.

[41] Ruan J.P., Chen L., Ma Z.L. (2019). Activation of spinal Extacellular Signal-Regulated Kinases and c-jun N-terminal kinase signaling pathways contributes to morphine-induced acute and chronic hyperalgesia in mice. J. Cell. Biochem..

[42] Lopes-Pires M.E., Frade-Guanaes J.O., Quinlan G.J. (2022). Clotting Dysfunction in Sepsis: A Role for ROS and Potential for Therapeutic Intervention. Antioxidants.

[43] Mittal M., Siddiqui M.R., Tran K., Reddy S.P., Malik A.B. (2014). Reactive oxygen species in inflammation and tissue injury. Antioxid Redox Signal.

[44] Jung J.S., Choi M.J., Lee Y.Y., Moon B.I., Park J.S., Kim H.S. (2017). Suppression of Lipopolysaccharide-Induced Neuroinflammation by Morin via MAPK, PI3K/Akt, and PKA/HO-1 Signaling Pathway Modulation. J. Agric. Food Chem..

[45] Sastre J., Pérez S., Sabater L., Rius-Pérez S. (2025). Redox signaling in the pancreas in health and disease. Physiol. Rev..

[46] Akhiani A.A., Martner A. (2022). Role of Phosphoinositide 3-Kinase in Regulation of NOX-Derived Reactive Oxygen Species in Cancer. Antioxidants.

[47] Jia J., Xu G., Zeng X., Patel V.B., Preedy V.R. (2022). The Biology of Morphine and Oxidative Stress. Handbook of Substance Misuse and Addictions: From Biology to Public Health.

[48] Doyle T., Bryant L., Muscoli C., Cuzzocrea S., Esposito E., Chen Z., Salvemini D. (2010). Spinal NADPH oxidase is a source of superoxide in the development of morphine-induced hyperalgesia and antinociceptive tolerance. Neurosci. Lett..

[49] Gessi S., Borea P.A., Bencivenni S., Fazzi D., Varani K., Merighi S. (2016). The activation of μ-opioid receptor potentiates LPS-induced NF-kB promoting an inflammatory phenotype in microglia. FEBS Lett..

[50] Rodriguez S., Sharma S., Tiarks G., Peterson Z., Jackson K., Thedens D., Wong A., Keffala-Gerhard D., Mahajan V.B., Ferguson P.J. (2024). Neuroprotective effects of naltrexone in a mouse model of post-traumatic seizures. Sci. Rep..

[51] Liu Y., Xu K., Xiang Y., Ma B., Li H., Li Y., Shi Y., Li S., Bai Y. (2023). Role of MCP-1 as an inflammatory biomarker in nephropathy. Front. Immunol..

[52] Kishi T. (2023). Clarification of hypertension mechanisms provided by the research of central circulatory regulation. Hypertens. Res..

[53] El-Naggar A.E., Helmy M.M., El-Gowilly S.M., El-Mas M.M. (2024). Suppression by central adenosine A3 receptors of the cholinergic defense against cardiovascular aberrations of sepsis: Role of PI3K/MAPKs/NFκB signaling. Front. Pharmacol..

[54] Ono-Saito N., Niki I., Hidaka H. (1999). H-series protein kinase inhibitors and potential clinical applications. Pharmacol. Ther..

[55] Sarkaria J.N., Tibbetts R.S., Busby E.C., Kennedy A.P., Hill D.E., Abraham R.T. (1998). Inhibition of phosphoinositide 3-kinase related kinases by the radiosensitizing agent wortmannin. Cancer Res..

[56] Park S.E., Song J.D., Kim K.M., Park Y.M., Kim N.D., Yoo Y.H., Park Y.C. (2007). Diphenyleneiodonium induces ROS-independent p53 expression and apoptosis in human RPE cells. FEBS Lett..

[57] Li H.X., Xu K., Chen S.L., Wang S.F., Li W.J. (2025). Current techniques for the treatment of spasticity and their effectiveness. EFORT Open Rev..

[58] Jiang R., Ai Z.-S., Jiang X., Yuan P., Liu D., Zhao Q.-H., He J., Wang L., Gomberg-Maitland M., Jing Z.-C. (2015). Intravenous fasudil improves in-hospital mortality of patients with right heart failure in severe pulmonary hypertension. Hypertens. Res..

[59] Ohbuchi M., Kimura T., Nishikawa T., Horiguchi T., Fukuda M., Masaki Y. (2018). Neuroprotective Effects of Fasudil, a Rho-Kinase Inhibitor, After Spinal Cord Ischemia and Reperfusion in Rats. Anesth. Analg..

[60] Nagel S., Genius J., Heiland S., Horstmann S., Gardner H., Wagner S. (2007). Diphenyleneiodonium and dimethylsulfoxide for treatment of reperfusion injury in cerebral ischemia of the rat. Brain Res..

[61] Seok Y.M., Azam M.A., Okamoto Y., Sato A., Yoshioka K., Maeda M., Kim I., Takuwa Y. (2010). Enhanced Ca2+-dependent activation of phosphoinositide 3-kinase class IIα isoform-Rho axis in blood vessels of spontaneously hypertensive rats. Hypertension.

[62] Murata Y., Fujiwara N., Seo J.H., Yan F., Liu X., Terasaki Y., Luo Y., Arai K., Ji X., Lo E.H. (2012). Delayed inhibition of c-Jun N-terminal kinase worsens outcomes after focal cerebral ischemia. J. Neurosci..

[63] Nguyen Thi P.A., Chen M.H., Li N., Zhuo X.J., Xie L. (2016). PD98059 Protects Brain against Cells Death Resulting from ROS/ERK Activation in a Cardiac Arrest Rat Model. Oxidative Med. Cell. Longev..

[64] El-Lakany M.A., Fouda M.A., El-Gowelli H.M., El-Gowilly S.M., El-Mas M.M. (2018). Gonadal hormone receptors underlie the resistance of female rats to inflammatory and cardiovascular complications of endotoxemia. Eur. J. Pharmacol..

[65] Yap J.Q., Nikouee A., Lau J.E., Walsh G., Zang Q.S. (2025). Mitochondria at the Heart of Sepsis: Mechanisms, Metabolism, and Sex Differences. Int. J. Mol. Sci..

[66] Bate S.T., Clark R.A. (2014). The Design and Statistical Analysis of Animal Experiments.

[67] El-Naggar A.E., Helmy M.M., El-Gowilly S.M., El-Mas M.M. (2023). Adenosine A1 receptors of the medullary solitary tract arbitrate the nicotine counteraction of neuroinflammation and cardiovascular dysfunction in septic rats. Sci. Rep..

[68] Gong W., Wen H. (2019). Sepsis Induced by Cecal Ligation and Puncture. Methods Mol. Biol..

[69] El-Mas M.M., Omar A.G., Helmy M.M., Mohy El-Din M.M. (2012). Crosstalk between central pathways of nitric oxide and carbon monoxide in the hypertensive action of cyclosporine. Neuropharmacology.

[70] El-Mas M.M., El-Gowelli H.M., Ghazal A.R., Harraz O.F., Mohy El-Din M.M. (2009). Facilitation of central imidazoline I(1)-site/extracellular signal-regulated kinase/p38 mitogen-activated protein kinase signalling mediates the hypotensive effect of ethanol in rats with acute renal failure. Br. J. Pharmacol..

[71] El-Mas M.M., Abdel-Rahman A.A. (1999). Role of the sympathetic control of vascular resistance in ethanol-clonidine hemodynamic interaction in SHRs. J. Cardiovasc. Pharmacol..

[72] El-Mas M.M., Abdel-Rahman A.A. (1999). Ethanol counteraction of I1-imidazoline but not alpha-2 adrenergic receptor-mediated reduction in vascular resistance in conscious spontaneously hypertensive rats. J. Pharmacol. Exp. Ther..

[73] Omar A.G., El-Mas M.M. (2004). Time-Domain Evaluation of Cyclosporine Interaction with Hemodynamic Variability in Rats. Cardiovasc. Drugs Ther..

[74] Paxinos G., Watson C. (1986). Atlas of the rat brain in stereotaxic coordinates.

[75] Baby S.M., Gruber R.B., Young A.P., MacFarlane P.M., Teppema L.J., Lewis S.J. (2018). Bilateral carotid sinus nerve transection exacerbates morphine-induced respiratory depression. Eur. J. Pharmacol..

[76] Bhargava H.N., Villar V.M. (1992). Pharmacodynamics and pharmacokinetics of intravenously administered morphine in spontaneously hypertensive and normotensive Wistar-Kyoto rats. J. Pharmacol. Exp. Ther..

[77] Kissin I., Kerr C.R., Smith L.R. (1983). Assessment of anaesthetic action of morphine and fentanyl in rats. Can. Anaesth. Soc. J..

[78] Dickenson A.H., Oliveras J.L., Besson J.M. (1979). Role of the nucleus raphe magnus in opiate analgesia as studied by the microinjection technique in the rat. Brain Res..

[79] Ibrahim K.S., El-Yazbi A.F., El-Gowelli H.M., El-Mas M.M. (2018). Heme oxygenase byproducts variably influences myocardial and autonomic dysfunctions induced by the cyclosporine/diclofenac regimen in female rats. Biomed Pharmacother..

[80] Sallam M.Y., El-Gowilly S.M., Abdel-Galil A.G., El-Mas M.M. (2016). Modulation by Central MAPKs/PI3K/sGc of the TNF-α/iNOS-dependent Hypotension and Compromised Cardiac Autonomic Control in Endotoxic Rats. J. Cardiovasc. Pharmacol..

[81] Kumar M., Bansal N. (2018). Fasudil hydrochloride ameliorates memory deficits in rat model of streptozotocin-induced Alzheimer’s disease: Involvement of PI3-kinase, eNOS and NFkappaB. Behav. Brain Res..

[82] Fujita M., Ando K., Nagae A., Fujita T. (2007). Sympathoexcitation by oxidative stress in the brain mediates arterial pressure elevation in salt-sensitive hypertension. Hypertension.

[83] Nassar N., Abdel-Rahman A.A. (2009). Brainstem adenosine A1 receptor signaling masks phosphorylated extracellular signal-regulated kinase 1/2-dependent hypotensive action of clonidine in conscious normotensive rats. J. Pharmacol. Exp. Ther..

