# Supplementary material for: Role of Central Inflammatory and Oxidative Pathways in the Morphine Exacerbation of Cardiovascular Effects of Sepsis in Rats
Source: Pharmaceuticals (Basel). 2025 Jun 12;18(6):882. doi: 10.3390/ph18060882 (PMC12195926; doi:10.3390/ph18060882)

Immunohistochemistry images for figure 6

Sham/Saline

- MCP1

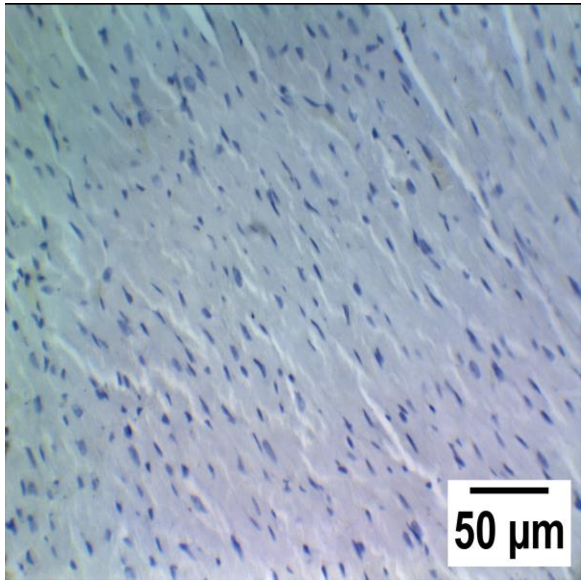

- TLR4

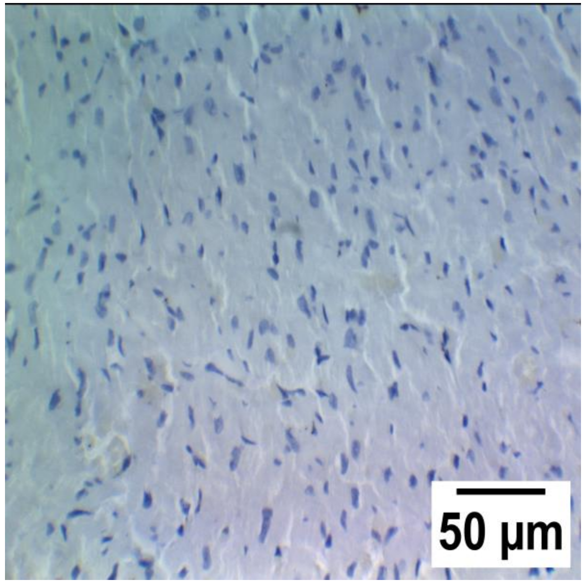

CLP/Saline

- MCP1

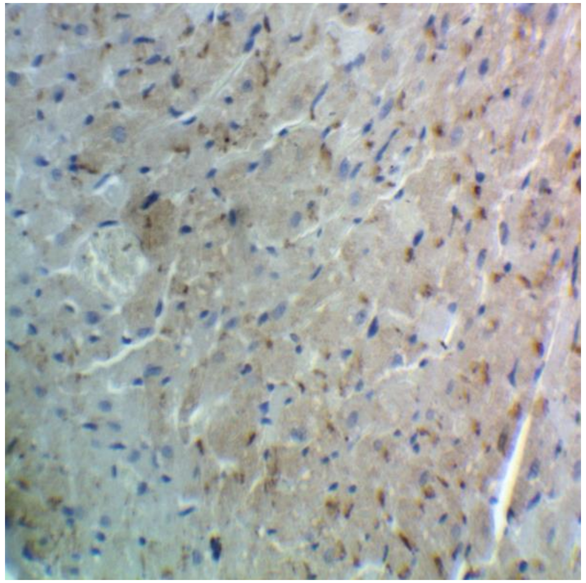

- TLR4

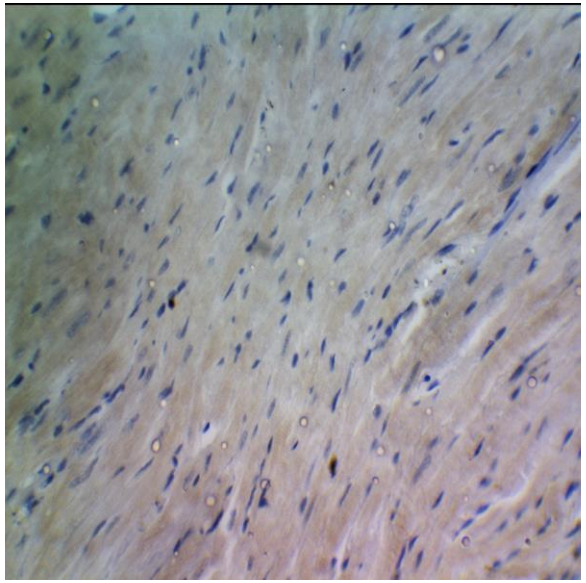

CLP/Morphine

- MCP1

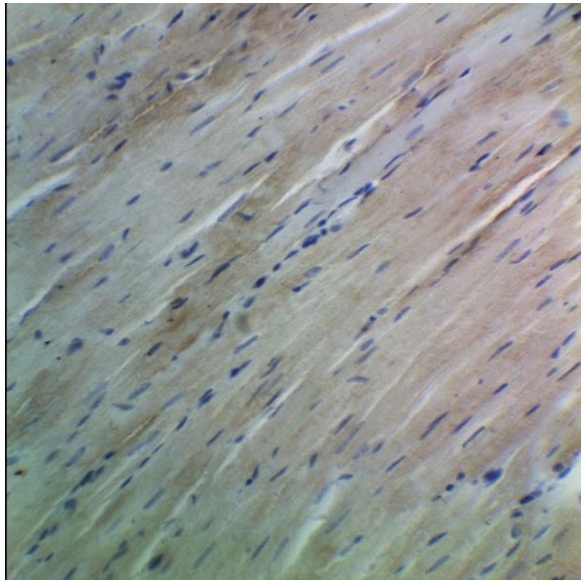

- TLR4

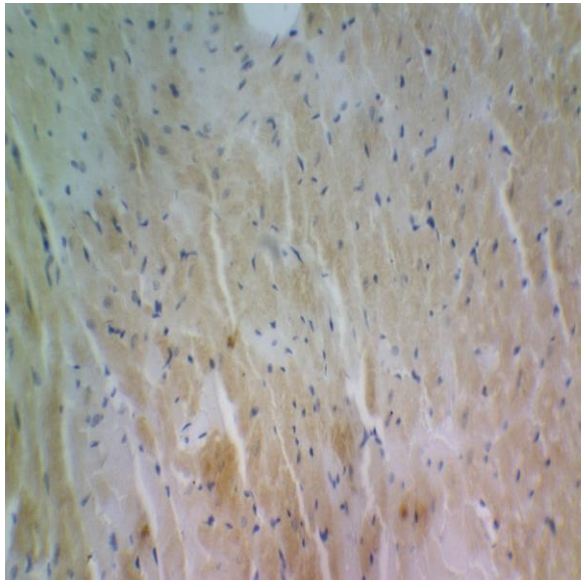

CLP/Naloxone/Morphine

- MCP1

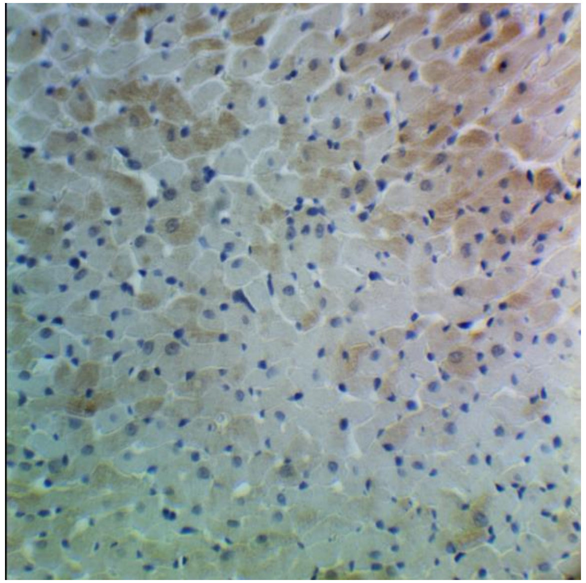

- TLR4

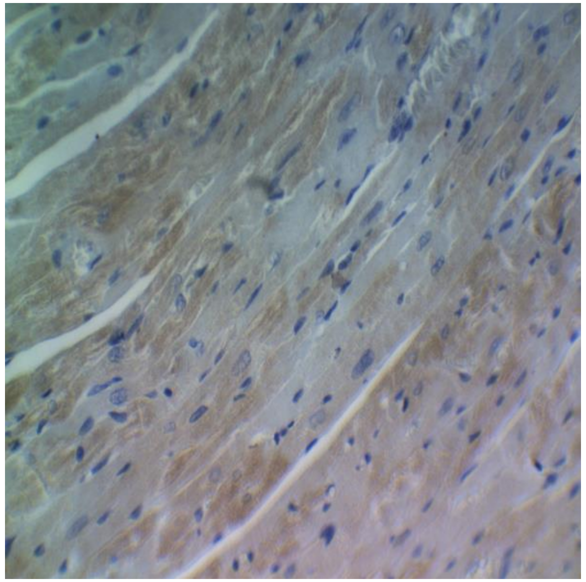

CLP/DPI/Morphine

- MCP1

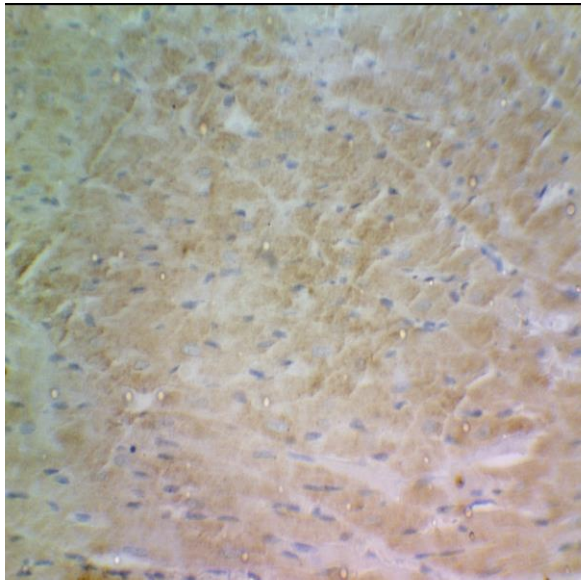

- TLR4

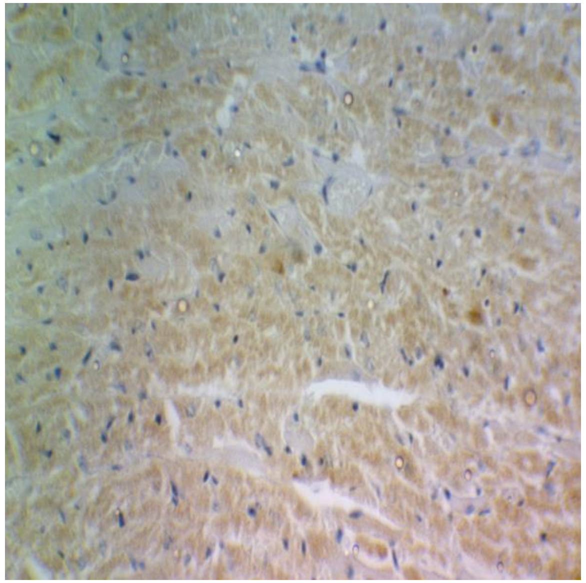

CLP/FAS/Morphine

- MCP1

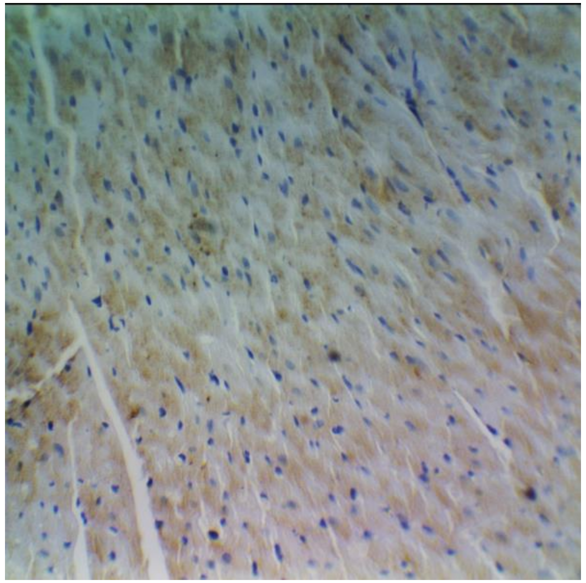

- TLR4

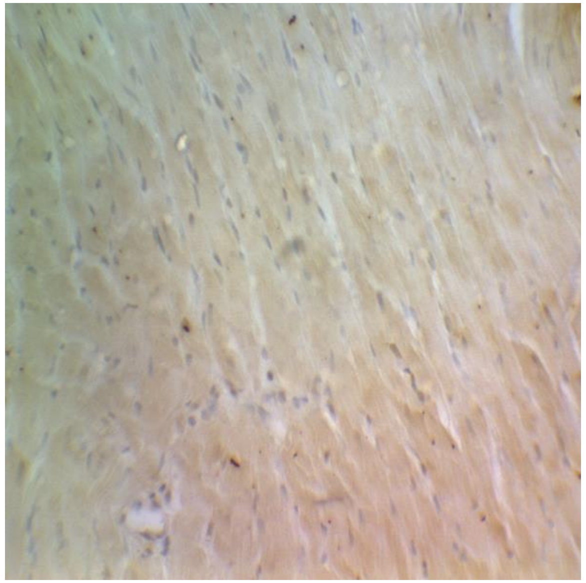

CLP/PD/Morphine

- MCP1

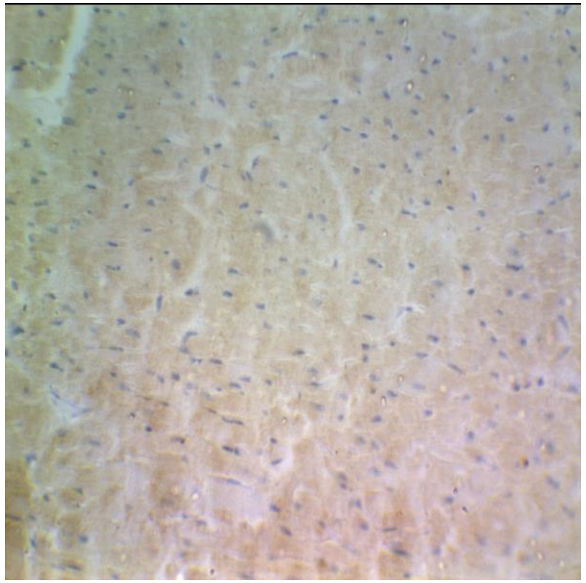

- TLR4

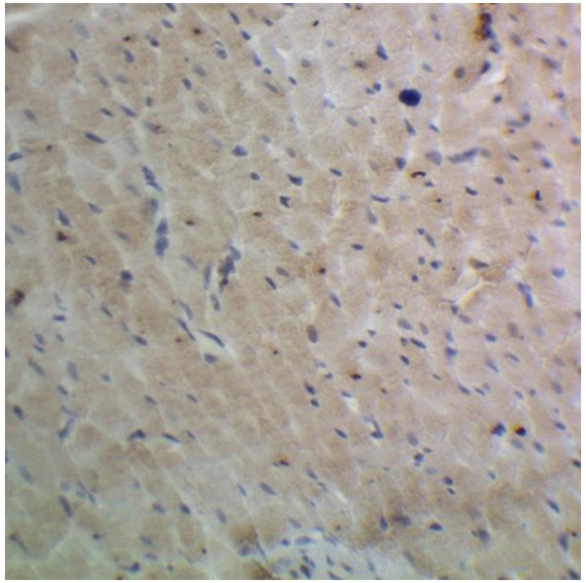

CLP/SP/Morphine

- MCP1

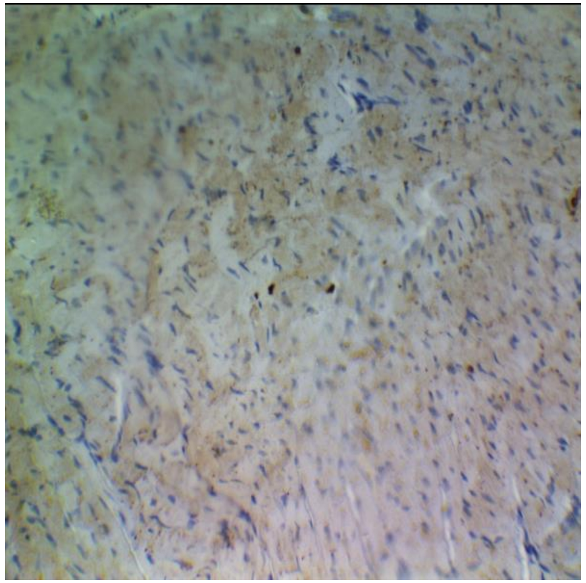

- TLR4

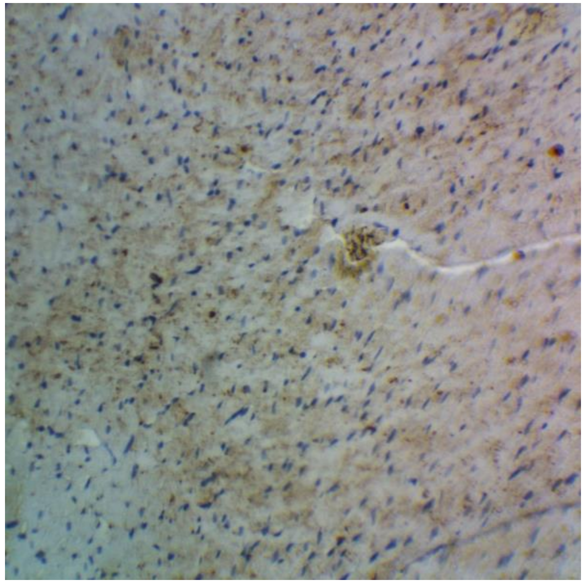

CLP/WM/Morphine

- MCP1

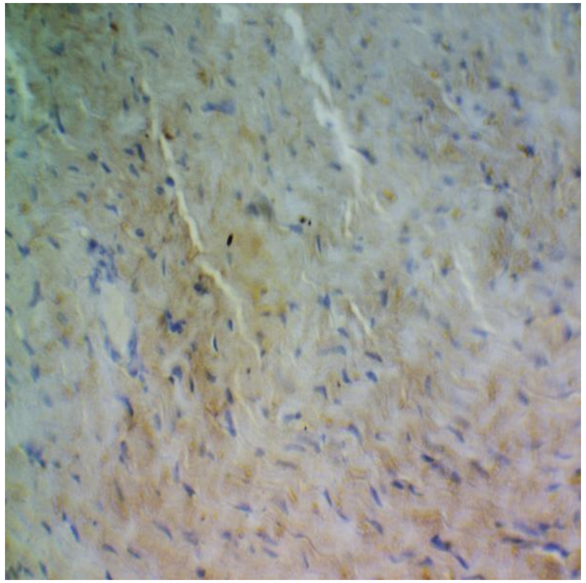

- TLR4

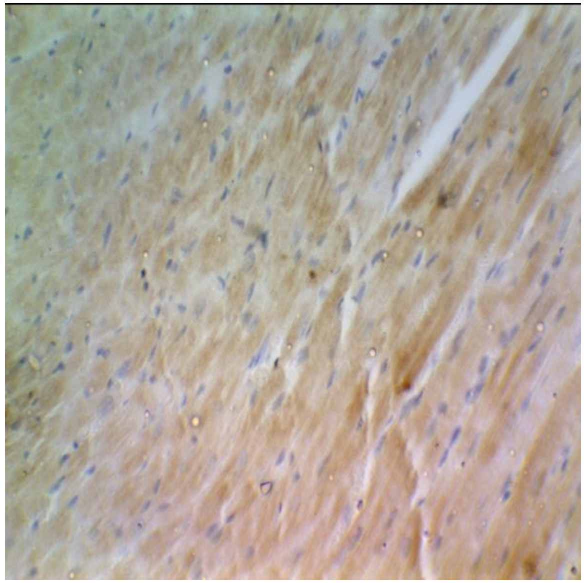

Supplement: Supplementary file 1 [file pharmaceuticals-18-00882-s001.zip › Supplemetary file S10, Uncropped Photos for cardiac immunohistochemical expression (Figure 6).pdf]
